# Health, cognitive, and psychosocial trajectories over nearly a decade as distal antecedents of awareness of age-related change in old age

**DOI:** 10.1093/geronb/gbag170

**Published:** 2026-08-20

**Authors:** Jana Koch, Laura Buchinger, Brooke Brady, Johanna Drewelies, Sandra Duezel, Ulman Lindenberger, Paolo Ghisletta, Nilam Ram, Ilja Demuth, Valentin Vetter, Kaarin J Anstey, Denis Gerstorf

**Affiliations:** School of Psychology, University of New South Wales, Sydney, New South Wales, Australia; Neuroscience Research Australia, Sydney, New South Wales, Australia; Department of Psychology, Humboldt University Berlin, Berlin, Germany; Socio–Economic Panel Study (SOEP), German Institute for Economic Research, DIW Berlin, Berlin, Germany; School of Psychology, University of New South Wales, Sydney, New South Wales, Australia; Neuroscience Research Australia, Sydney, New South Wales, Australia; Center for Environmental Neuroscience, Max Planck Institute for Human Development, Berlin, Germany; Charité—Universitätsmedizin Berlin, Berlin, Germany; Max Planck UCL Centre for Computational Psychiatry and Ageing Research, Berlin, Germany and London, United Kingdom; Center for Lifespan Psychology, Max Planck Institute for Human Development, Berlin, Germany; Department of Psychology, University of Geneva, Geneva, Switzerland; Department of Communication, Stanford University, Palo Alto, California, United States; Department of Psychology, Stanford University, Palo Alto, California, United States; Charité—Universitätsmedizin Berlin, Berlin, Germany; Charité—Universitätsmedizin Berlin, Berlin, Germany; School of Psychology, University of New South Wales, Sydney, New South Wales, Australia; Neuroscience Research Australia, Sydney, New South Wales, Australia; Department of Psychology, Humboldt University Berlin, Berlin, Germany; Socio–Economic Panel Study (SOEP), German Institute for Economic Research, DIW Berlin, Berlin, Germany; (Psychological Sciences Section)

**Keywords:** Health trajectories, Longitudinal, Morbidity, Cognitive impairment, Loneliness

## Abstract

**Objectives:**

Awareness of age-related change (AARC) reflects how individuals perceive age-related gains and losses, with positive perceptions consistently predicting better health and well-being. The Awareness of Age-Related Change theoretical framework has long highlighted physical and psychosocial health as distal antecedents of AARC, but their actual importance remains poorly understood. In this preregistered study, we empirically test this key model component.

**Methods:**

We operationalize distal antecedents as 9-year trajectories in physical health (morbidity, grip strength, hearing, vision), cognitive functioning (recall, processing speed, working memory), and self-reported loneliness. We apply structural equation modeling to 3-wave longitudinal data from the Berlin Aging Study II (*N *= 753, aged 61–81 at baseline, 49% female) and test if these trajectories predict late-life AARC.

**Results:**

Older adults with better baseline recall and grip strength reported fewer AARC losses, whereas those with higher baseline loneliness perceived more losses and fewer gains. Steeper declines in recall and stronger increases in morbidity were associated with heightened awareness of losses. Processing speed, working memory, hearing, and vision were not associated with AARC.

**Discussion:**

Our results suggest several refinements of the Awareness of Age-Related Change framework: (a) within a given construct space (e.g., cognition), some dimensions (e.g., recall) are more strongly associated with perceiving age-related losses than others (e.g., processing speed), (b) long-term trajectories of recall memory and morbidity (rather than status alone) are particularly relevant for loss-focused awareness of aging, and (c) higher levels of loneliness are most consistently associated with awareness of age-related gains.

Subjective views of aging reflect how individuals perceive age-related changes in health, cognition, and everyday functioning (Shrira et al., 2022). They matter because individuals with more positive views of aging engage more frequently in preventative health behaviors (Levy, 2009) and show better psychological and physical health, including longer survival (Sabatini et al., 2020a; Westerhof et al., 2023). Recent studies in this area have increasingly sought to identify which factors predict whether individuals maintain positive or develop negative views of aging (e.g., Diehl et al., 2021b; Kornadt et al., 2025; Sabatini et al., 2022; Schönstein et al., 2021). However, most have examined antecedents of unidimensional constructs that position age views along a single continuum from negative to positive. Prior work has also often been limited by small samples and relied on simple change-score predictors rather than modeling change across multiple waves. Leveraging 9-year longitudinal data, here, we test how both levels of and changes in multiple indicators of health, cognition, and psychosocial functioning predict awareness of age-related gains and losses, thereby providing a direct empirical test of the distal antecedent component of the Awareness of Age-Related Change framework (Diehl & Wahl, 2010).

## Theoretical foundations of awareness of age-related change

Awareness of age-related change (AARC) refers to “experiences that make a person aware that their behavior, level of performance, or ways of experiencing their life have changed as a consequence of having grown older” (Diehl & Wahl, 2010, p. 340). Following tenets of lifespan development theory (Baltes, 1987), AARC captures positive and negative experiences (termed AARC-gains and AARC-losses). These can co-occur across life domains, including health and physical functioning, cognitive functioning, interpersonal relationships, social-cognitive/social-emotional functioning, and lifestyle and engagement (Kaspar et al., 2019).

Although AARC is relevant across all of adulthood, it becomes more prominent in later life when accumulated changes make awareness of losses more likely. Research shows that throughout midlife, AARC-losses remain relatively stable but increase after age 65 (Diehl et al., 2021b; Kaspar et al., 2023). According to the Awareness of Age-Related Change framework (Diehl & Wahl, 2010), these developmental trends reflect the combined influence of distal and proximal antecedents, which can differ for AARC-losses and AARC-gains. Distal antecedents represent long-term influences that contribute to an individual’s sense of aging, including sociodemographic, psychological, and health-related factors. Proximal antecedents (not examined here) reflect immediate or situational triggers (e.g., major life events) that make individuals aware of their current stage of life.

Theoretically, changes in health, cognition, and functioning can only be reflected in AARC when they are consciously recognized; thus, AARC is understood as a form of conscious self-knowledge about one’s own aging (Diehl & Wahl, 2010). Consistent with this idea, subjective appraisals of health are strongly associated with views of aging (Kaspar et al., 2019; Schönstein et al., 2021; Siebert et al., 2020). However, much less is known about the role of objectively measured markers for AARC. Given that objective age-related changes are not always subjectively perceived (e.g., correlations between objective and subjective memory decline are often only small to moderate; Hertzog et al., 2018), not every objectively measured change in functioning is likely to be interpreted as age-related, and therefore reflected in one’s AARC. The present study takes advantage of a rich set of longitudinal data on physical health and performance-based measures that are rarely available in studies on subjective views of aging. These data allow us to link long-term trajectories in health, cognition, and psychosocial functioning to AARC, thereby anchoring it more firmly in objective developmental processes and allowing us to empirically test which trajectories feed into one’s aging awareness.

## Empirical evidence on distal antecedents of awareness of age-related change

Drawing on Diehl and Wahl’s (2010) conceptual framework, we examined three domains of distal antecedents: (a) objectively measured markers of physical health (i.e., morbidity, grip strength, hearing loss, and vision acuity); (b) performance-based markers of cognitive functioning (i.e., recall, processing speed, and working memory); and (c) psychosocial well-being (i.e., self-reported loneliness).

### Physical health

Prior research showed that poorer health status is associated with more negative views of aging (Kleinspehn-Ammerlahn et al., 2008; Sabatini et al., 2022; Schönstein et al., 2021; Stephan et al., 2015). The accumulation of diseases may act as everyday signals of aging and reinforce awareness of one’s place on the aging continuum. Importantly, associations between morbidity and views of aging are not contingent on the severity or terminal nature of a condition (Schönstein et al., 2021). This suggests that the mere presence of disease is sufficient to heighten AARC-losses, and it stands to reason that increases in morbidity over time may additionally contribute.

Grip strength as a performance-based marker of upper-body muscle strength is considered a key reflection of overall physical health (Vaishya et al., 2024). Prior work has shown that better grip strength is linked to better performance on everyday tasks and to greater health-related quality of life (Taekema et al., 2010). This suggests that grip strength may also be relevant for how individuals evaluate their own aging. In line with this, Stephan et al. (2015) showed that better grip strength was associated with younger subjective age in cross-sectional data from the Health and Retirement Study.

Sensory decline, particularly hearing and vision loss, is among the most common conditions in later life and is often regarded as a first marker of aging that reinforces negative age stereotypes and stigma (World Health Organization, 2023). Both sorts of sensory impairments significantly affect daily functioning, social relationships (Gopinath et al., 2012), and cognitive health (Loughrey et al., 2018). This predisposes them as likely risk factors for loss-focused aging experiences. In line with this, longitudinal evidence shows that poorer self-reported hearing is associated with more AARC-losses (but not fewer AARC-gains) in physical health and cognitive functioning domains (Wettstein et al., 2023). Other studies have similarly linked hearing loss to less favorable views of aging (Koch et al., 2025a). We are not aware of any study that investigated predictive effects of visual acuity for AARC.

### Cognitive functioning

Cognitive functioning plays a prominent role in research on views of aging because it carries strong cultural and stereotypical connotations and directly impacts older adults’ daily functioning (Heckhausen et al., 1989; Kornadt & Rothermund, 2011). In a sample of 103 older adults aged 61 and above, 20-year declines in processing speed (measured using simple baseline-to-follow-up change scores) predicted heightened AARC-losses and fewer AARC-gains (Sabatini et al., 2022). However, other studies found no association between cognitive decline and views of aging (Schönstein et al., 2021; Siebert et al., 2020). Therefore, studies that apply contemporary longitudinal modeling to more than two waves of data obtained from large samples of older adults are needed before firm conclusions about the role of cognitive trajectories can be drawn.

### Psychosocial functioning

Psychosocial well-being, and social connectedness in particular, is a major correlate of views of aging, often more predictive than performance-based indicators (Schönstein et al., 2021). Kornadt and colleagues (2025) showed that loneliness was associated with feeling subjectively older (a marker of more negative aging perceptions) and predicted steeper increases in subjective age over time. Social contact frequency typically declines in later life (Wrzus et al., 2013), but social connectedness remains a critical psychosocial resource for resilience. According to the stress-buffering hypothesis (Cohen & Wills, 1985), age-related challenges are more likely to be perceived as losses rather than manageable changes when social resources are lacking.

## The current study

The current pre-registered study examined how 9-year trajectories of physical health (i.e., morbidity, grip strength, hearing function, vision acuity), cognitive functioning (i.e., recall, working memory, processing speed), and psychosocial well-being (i.e., loneliness) predict AARC-losses and AARC-gains. To build on prior studies in this space, we leveraged three-wave, longitudinal data from a large sample of older adults (60+) in the Berlin Aging Study II (BASE-II; Gerstorf, 2016) that allowed us to model these long-term developmental patterns across a comprehensive set of eight health indicators using structural equation modeling.

Based on the theory and prior research outlined above, we derived the following preregistered hypotheses: Drawing on the AARC framework (Diehl & Wahl, 2010) and prior empirical work (e.g., Kaspar et al., 2022), we hypothesized that poorer average health, cognitive, and psychosocial functioning and steeper decline in these three categories of functioning over time are associated with more AARC-losses and fewer AARC-gains (H1). On the basis of Diehl and Wahl’s (2010) proposition that negative age-related experiences occur more often in the physical health and cognitive domains than in the social or emotional domain, and the lifespan developmental premise of a more loss-dominated gain-loss ratio in old age (Baltes, 1987), we expected physical health and cognitive functioning to show stronger associations with AARC-losses than with AARC-gains (e.g., Kaspar et al., 2022; Sabatini et al., 2020a) (H2). A central premise of the AARC framework is that perceived age-related change presupposes conscious awareness of changes in one’s behavior, level of performance, or ways of experiencing life (Diehl & Wahl, 2010). Following from this premise, it stands to reason that consciously experienced states such as feelings of loneliness are more readily incorporated into an individual’s views of aging than functional changes that may go unnoticed in everyday life. Accordingly, we hypothesized that loneliness will show stronger associations with both AARC-losses and AARC-gains than the objectively measured markers of physical health and cognitive functioning (H3).

We control for demographics (age at baseline, sex, education), acknowledging that AARC-losses increase with age (Kaspar et al., 2023), while higher education and female sex are associated with more favorable perceptions of aging (English et al., 2019; Miche et al., 2014; Rupprecht et al., 2022).

## Method

### Participants and procedures

We used data from BASE-II, a longitudinal, multi-institutional, and interdisciplinary study initiated in 2009 with a baseline cohort of 1,671 older adults from the greater metropolitan area of Berlin, Germany (for additional information, see: Bertram et al., 2014; Demuth et al., 2021; Gerstorf, 2016). Medical assessments were conducted by trained physicians as part of two half-day visits to the Charité University Hospital and included a detailed medical history, physician-administered physical examinations, and performance-based measures such as grip strength, hearing, and vision. Cognitive testing was carried out by trained scientific staff on computers in group sessions of three to six participants. Psychosocial variables, including AARC and loneliness, were obtained as part of take-home questionnaires.

Here, we used data from three waves of BASE-II, assessed in 2012–2014 (T1), 2018/19 (T2), and 2022/23 (T3). Participants were included if they completed the AARC measure at T3 with less than 20% missing data (*n *= 759). Mean age at baseline was *M *= 69.87 (*SD *= 3.65, range 60.7–80.8) years; 49.3% were women, and average education duration was *M *= 14.46 (*SD *= 2.85) years.

Ethical approval for the BASE-II was granted by the ethics committee of the Charité-Universitätsmedizin and the Max Planck Institute for Human Development, Berlin, Germany (EA2/029/09, EA2/144/16, and EA2/224/21). Ethical approval for the secondary analysis was obtained from the University of New South Wales Human Research Ethics Committee (iRECS8874). The study was preregistered on OSF (https://osf.io/j3sha).

### Measures

#### Awareness of age-related change

At T3, participants completed the 10-item short form of the AARC scale (Kaspar et al., 2019). Five items capture gain-related experiences (e.g., “With increasing age, I realize that I appreciate relationships and people much more”) and five capture loss-related experiences (e.g., “With increasing age, I realize that I feel more dependent on the help of others”). Participants rated items on a 5-point Likert scale (1 = *Not at all*, to 5 = *Very much*). Sum scores for AARC-gains and AARC-losses were computed. Internal consistency (McDonald’s *ω*) was 0.80 and 0.70 for AARC-losses and AARC-gains, respectively. The AARC scale was originally developed as a 50-item questionnaire (Brothers et al., 2019), with a 10-item short form developed for inclusion in larger studies, and the factor structure, measurement invariance, and concurrent and discriminant validity of the AARC 10-item short form have been established (Kaspar et al., 2019). However, we note that the AARC- internal consistency for the AARC-gains scale is relatively low in our sample, likely because of the brief five-item nature of the measure. Comparable internal consistencies of the AARC-gains scale have been reported in previous research, including McDonald’s *ω* = .68 in Kaspar et al. (2022), and Cronbach’s *α =* .77 in Sabatini et al. (2020b).

#### Physical health

##### Morbidity

Morbidity was measured at T1–T3 with an index adapted from the Charlson Comorbidity Index, representing a weighted classification of common diseases (Meyer et al., 2016). Diagnoses were based on clinical examinations in addition to participant-reported medical histories. Higher scores represent greater morbidity.

##### Grip strength

Grip strength (collected T1–T3) was assessed as the mean strength in kilograms exerted across six dynamometer trials, three per hand. Higher scores indicate greater strength.

##### Sensory functioning

Hearing was measured at baseline (T1) using pure-tone audiometric air conduction testing on both ears. The pure-tone average (0.1, 1, 2, and 4 kHz) of the better ear was used. Higher values indicate worse hearing. Visual acuity was measured at baseline (T1) for each eye using a decimal chart with numbers as optotypes at 4 m. Higher scores indicate better distance vision.

#### Cognitive functioning

All cognitive functions were assessed at T1–T3. Additional information on the tests can be found in Supplementary Section A.

##### Recall

In the Verbal Learning and Memory Test (VLMT), participants listened to 15 words in the same order across five learning trials and typed as many as they could recall after each trial. The sum of correctly recalled words across all trials was used as an immediate recall indicator. The 15 words to be remembered were the same across all waves.

##### Working memory

In the Number-N-Back (NNB) task, 30 sequences of three single-digit numbers (ranging from 0 to 9) in three horizontally aligned cells were presented. Participants made two-choice decisions about whether the current stimulus matched the one presented three steps earlier. After four practice trials (30 runs each), participants completed six test trials (30 runs each). The mean accuracy across all stimuli was used as the performance score.

##### Processing speed

The Digit Symbol Substitution Test (DSST), a subtest from the Wechsler Adult Intelligence Scale-Revised (Wechsler, 1955), required participants to match symbols to digits based on a provided key, under time pressure. The test score is derived from the number of correct symbol entries, with a penalty for wrong answers (score = total − wrong). Higher scores represent better processing speed.

#### Psychosocial functioning

Loneliness (collected T1–T3) was measured with seven items from the UCLA Loneliness Scale (Russell et al., 1984). Participants were asked to rate statements such as “I lack companionship” on a 5-point Likert scale (1 = *does not apply to me at all*, to 5 = *applies very well to me*). The seven items were summed for an overall loneliness score, with higher scores indicating a higher level of loneliness. Internal consistency (Cronbach’s *α*) of the loneliness scale ranged from .83 to .86 across the three waves.

#### Sociodemographic correlates

We included chronological age (at baseline), sex (women = 1, men = 0), and total number of years of formal education (at baseline). For the analysis, chronological age was centered at 75, and sex and education were grand mean-centered.

### Statistical procedure

All predictor and outcome variables were T-standardized based on baseline values. For cases with <20% missing values on the AARC scale (*n *= 29), missing responses were imputed using sample means. Structural equation models were estimated in Mplus; data preparation, descriptives, and visualization were done in R (R Core Team, 2025).

First, we fitted a series of latent growth curve models (LGCM) for each longitudinal predictor (morbidity, grip strength, DSST, VLMT, NNB, loneliness) to estimate mean-level change and variability in change across individuals (Preacher et al., 2008). Time was coded as average years since baseline: T1 = 0 (2012–2014), T2 = 5.406 (2018), T3 = 9.360 (2022). Residual variance was constrained to be equal across measurement occasions. Missing data were handled using the full information maximum likelihood (FIML) with robust standard errors. Model fit was evaluated using the sample size-adjusted Bayesian Information Criterion (BIC) and global fit indices (χ^2^, CFI, RMSEA, SRMR). We compared models with only linear and both linear and quadratic slope factors to determine the functional form of the trajectories. For all six predictors, univariate growth models with freely estimated variances of both the linear and quadratic slope factors did not converge. Therefore, we retained both slopes but fixed the quadratic slope variance to zero.

Second, we estimated six prediction models (one per longitudinal predictor) in which AARC-losses and AARC-gains (T3) were regressed on the intercept and slope factors. To account for practice effects on the cognitive tests, we included latent retest factors. For DSST and VLMT, loadings were fixed at 0, 1, and 1; for NNB, this specification did not converge, and a linear pattern (0, 1, 2) was used instead. To calculate the standardized effect sizes, we multiplied the unstandardized regression coefficient by the deviation of the predictor (reflecting a 1-*SD* increase) and divided it by the standard deviation of the AARC outcome.

For hearing and vision, assessed only at baseline, we specified two prediction models treating them as time-invariant predictors of AARC. Chronological age, sex, and education were included as time-invariant covariates in all models. Statistical tests with *p* < .05 were interpreted as evidence for statistical significance.

## Results

On average, participants reported more gains (*M *= 18.2, *SD *= 3.13) than losses (*M *= 12.8, *SD *= 3.49) at T3. AARC-gains were relatively stable across age, whereas AARC-losses were higher in older ages (see Figure 1), amounting to a 1.5 standard deviation (*SD*) difference between those aged 70 and those aged 90. Descriptive statistics are presented in Table 1 and baseline intercorrelations in Table S1.

**Figure 1:**
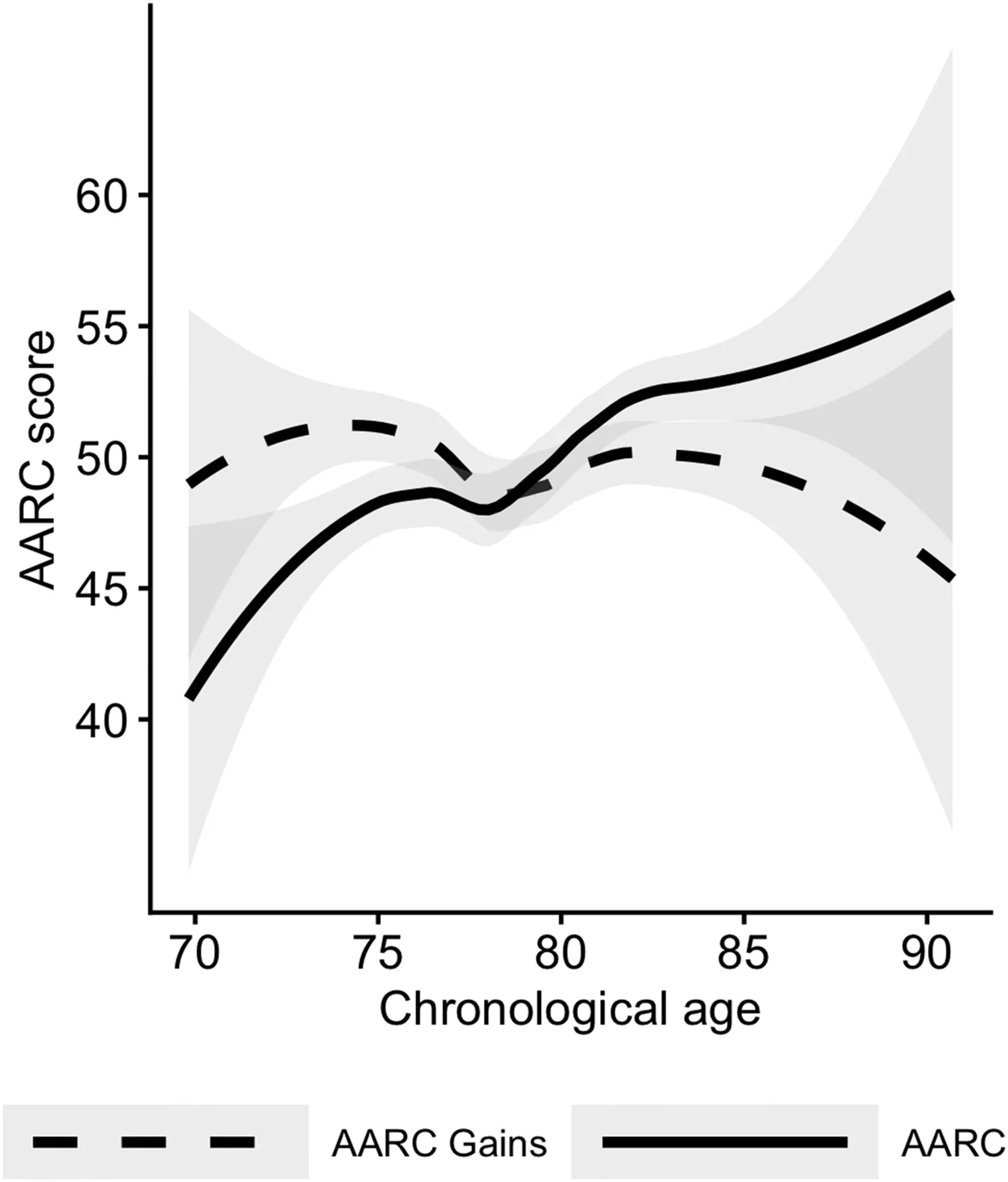
Distribution of awareness of age-related gains and losses at T3 across chronological age. AARC = awareness of age-related change. Awareness of age-related gains (dashed line) and losses (solid lines) are displayed as *t*-scores (*M* = 50, *SD* = 10) to facilitate interpretation of age differences. For example, awareness of age-related losses is higher at age 90 with a mean of around 55 compared to age 70 with a mean of around 40, reflecting a cross-sectional age difference of about 1.5 *SDs* over 20 years. Curves were smoothed using locally estimated scatterplot smoothing (LOESS).

**Table 1:** Sample characteristics and descriptive statistics.

Variables	T1		T2		T3	
	*n*	% or *M* (*SD*)	*n*	% or *M* (*SD*)	*n*	% or *M* (*SD*)
**Demographics**						
Age, years	743	69.9 (3.65)	693	75.3 (3.77)	759	79.2 (3.53)
Education, years	755	14.5 (2.85)				
Sex, % female	374	49.3				
**Outcome variables**						
Awareness of age-related losses	–			–	759	12.8 (3.49)
Awareness of age-related gains	–			–	759	18.2 (3.13)
**Longitudinal predictor variables**						
Cognitive functioning						
Number N–Back Task	742	0.76 (0.18)	684	0.62 (0.20)	755	0.80 (0.20)
Digit Symbol Task	703	44.3 (10.04)	670	43.9 (10.20)	735	40.3 (10.45)
Verbal Learning and Memory Task	740	45.2 (12.68)	600	45.2 (12.14)	745	41.9 (13.50)
Physical functioning						
Grip strength	613	35.1 (9.63)	695	28 (9.12)	295	28.3 (8.82)
Morbidity	683	0.98 (1.18)	692	1.7 (1.60)	370	2.0 (1.72)
Sensory functioning						
Hearing function	357	19.1 (8.43)		–		–
Visual acuity	528	1.0 (0.27)		–		–
Loneliness	743	1.5 (0.62)	644	1.6 (0.62)	756	1.7 (0.69)

*Note.* Descriptive statistics are presented in raw units for interpretability. For all latent growth and prediction models, these variables were rescaled to *t*-scores.

### Univariate latent growth curve models

Results from the unconditional growth models indicated significant change across the 9-year period for all investigated health and performance domains (see Table S2). On average, morbidity increased, whereas grip strength, VLMT, NNB, and DSST declined across the 9-year period. Loneliness showed no significant mean-level change. We observed significant interindividual differences in change for morbidity, VLMT, and NNB, with linear slope variances ranging from 0.39 to 1.20, but found no significant slope variance for loneliness, grip strength, or DSST (Table S2). Interindividual differences in development (or lack thereof) are visualized for all predictor variables in Figure S1.

### Prediction models

Parameter estimates of the series of prediction models are reported in Table 2. Figure 2 visualizes significant predictions of AARC-losses and AARC-gains from latent growth curve models. In partial support of H1, we found that some levels and trajectories of physical health, cognitive, and psychosocial functioning predicted AARC-losses and AARC-gains at T3. For AARC-losses, we found that lower baseline recall (VLMT; *b* = –0.16, CI [– 0.29, –0.03], *p* = .013), weaker baseline grip strength (*b* = –0.20, CI [–0.34, –0.05] *p* = .009), and higher baseline loneliness (*b *= 0.35, CI [0.24, 0.46], *p* < .001) each predicted greater AARC-losses at T3. Baseline levels of morbidity, processing speed, and working memory did not predict AARC-losses, nor did performance-based assessments of hearing or vision (see Table 3). Regarding change trajectories, steeper decline in recall (VLMT, *b* = –2.80, CI [–5.16, –0.44], *p* = .020) and steeper increase in morbidity (*b *= 1.11, CI [0.14, 2.07], *p* = .024) each predicted more AARC-losses. Standardized, a 1-*SD* decline in recall predicted a 0.18-*SD* increase in AARC-losses, and a 1-*SD* increase in morbidity predicted a 0.12-*SD* increase. Declines in processing speed and working memory did not predict greater AARC-losses. For loneliness and grip strength, we were unable to model and regress the slope factors on AARC outcomes because of limited interindividual variability in rate of change. For AARC-gains, the only significant predictor was baseline loneliness, such that higher loneliness was associated with lower AARC-gains at T3 (*b* = –0.19, CI [–0.29, –0.09], *p* < .001). These effects translate to a 0.16-*SD* decrease in AARC-gains per 1-*SD* increase in loneliness. None of the other indicators of physical health or cognitive functioning, or their trajectories, were significantly related to AARC-gains. These results also support H2, in that physical health and cognitive functioning showed stronger associations with AARC-losses than with AARC-gains.

**Figure 2:**
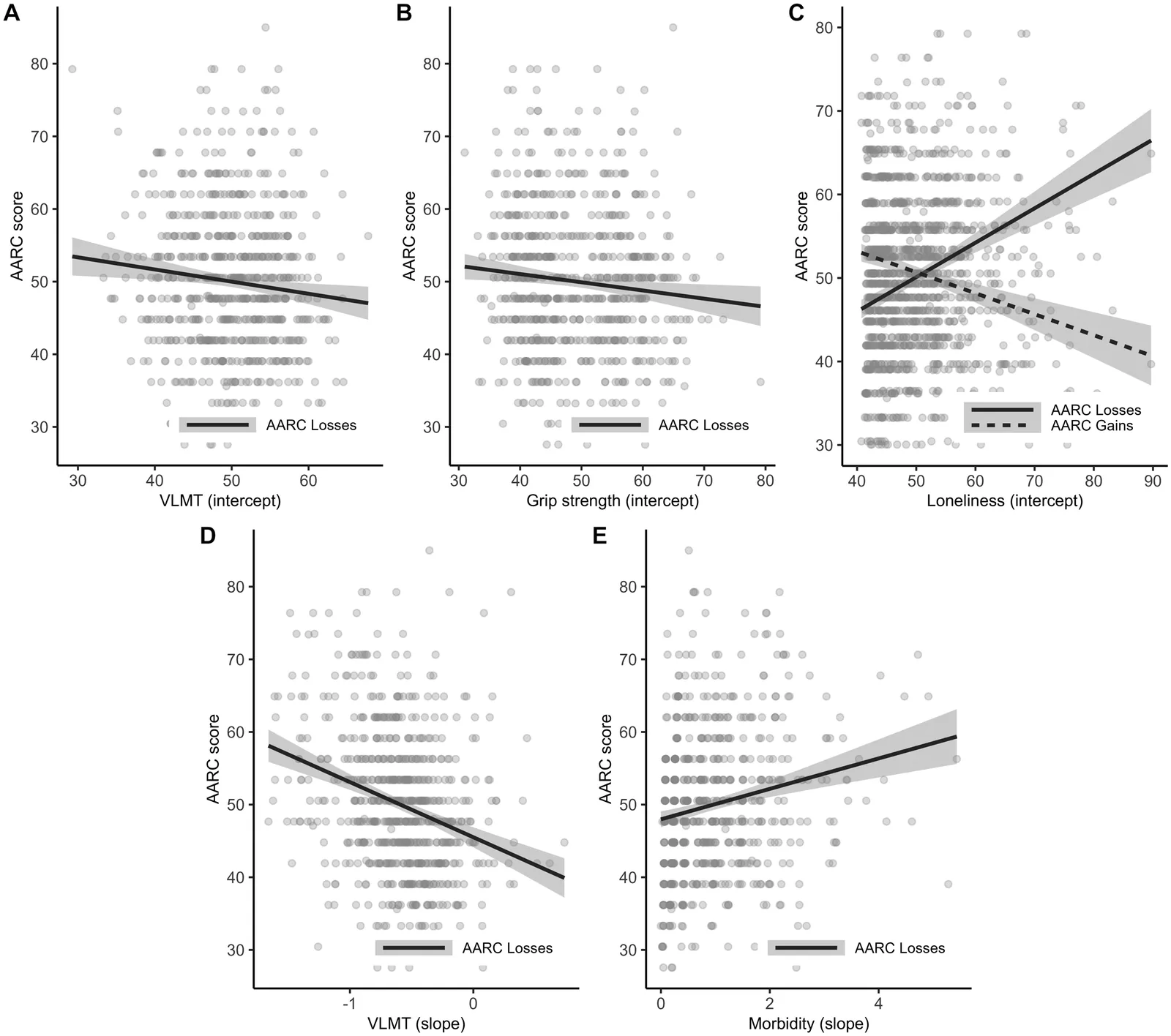
Visualization of latent intercept and slope estimates from growth curve models predicting awareness of age-related gains and losses. AARC = awareness of age-related change; VLMT = Verbal Learning and Memory Task. Significant predictions of AARC, scaled as *t*-scores, *M* = 50, *SD* = 10. Panels A–C show associations between the baseline-level latent intercept factors of VLMT (recall), grip strength, and loneliness, respectively, and AARC outcomes. Panels D and E show associations between the latent slope factors (change over time) of VLMT and morbidity, respectively, and AARC outcomes.

**Table 2:** Linear latent growth curve models (time-in-study) of health, cognition, and loneliness predicting awareness of age-related gains and losses.

Variables	VLMT		NNB		Digit symbol		Morbidity		Grip strength		Loneliness	
	Est.	*SE*	Est.	*SE*	Est.	*SE*	Est.	*SE*	Est.	*SE*	Est.	*SE*
**Growth model**												
Mean intercept	49.98*	0.38	50.03*	0.38	49.88*	0.39	50.05*	0.39	49.19*	0.40	49.95*	0.37
Mean slope	–0.59*	0.09	–12.80*	0.52	–0.86*	0.07	0.97*	0.05	–1.0*	0.03	0.20*	0.04
Retest	3.38*	0.76	61.13*	2.55	4.22*	0.55	–	–	–	–	–	–
Var. intercept	54.80*	5.14	53.40*	6.28	76.35*	5.61	86.44*	9.47	82.08*	3.9	68.11*	6.96
Var. slope	0.41*	0.10	0.40*	0.15	0.12	0.07	1.20*	0.16	0.00	0.00	0.00	0.00
Cov. intercept, slope	–1.02	0.56	–2.09*	0.79	0.04	0.39	2.71*	0.66	0.00	0.00	0.00	0.00
Residual variance	39.99*	2.98	69.80*	4.48	22.91*	2.96	17.28*	1.65	16.53*	1.01	40.44*	3.38
**Prediction model**												
Sex	0.001	–	0.001	–	0.001	–	0.001	–	0.001	–	0.001	–
Education	–0.01	–	–0.01	–	–0.01	–	–0.01	–	–0.01	–	–0.01	–
Age	–5.13	–	–5.13	–	–5.13	–	–5.13	–	–5.13	–	–5.13	–
Intercept AARC-gains	48.51*	3.43	88.60*	23.7	51.95*	4.97	46.87*	2.47	53.25*	3.68	58.83*	2.66
Intercept AARC-losses	58.27*	3.32	20.38	20.2	48.35*	6.71	46.80*	2.58	61.55*	3.68	34.74*	2.87
Residual variance, gains	99.51*	6.41	95.84*	6.64	97.03*	7.19	99.34*	6.33	99.31*	6.37	97.06*	6.51
Residual variance, losses	94.30*	5.95	95.32*	5.95	90.56*	8.41	95.23*	5.53	96.66*	5.55	89.59*	5.29
Cov. gains, losses	6.49	4.25	9.21*	4.67	10.48*	5.02	5.72	4.32	5.73	4.29	10.74*	4.17
AARC-gains on												
Sex	1.09	0.79	0.94	0.78	1.03	0.79	1.21	0.78	–0.07	1.31	0.94	0.77
Education	0.22	0.14	0.26	0.14	0.21	0.14	0.23	0.14	0.23	0.14	0.19	0.14
Age	–0.15	0.10	–0.14	0.10	–0.11	0.10	–0.16	0.10	–0.18	0.10	–0.14	0.10
Intercept	0.02	0.07	0.03	0.08	0.03	0.05	0.05	0.05	–0.09	0.08	–0.19*	0.05
Linear slope	0.15	1.03	3.19	1.90	4.60	3.72	0.03	0.47	–	–	–	–
AARC-losses on												
Sex	0.50	0.78	0.17	0.77	0.21	0.78	0.18	0.76	–2.74*	1.32	0.34	0.74
Education	0.12	0.14	0.01	0.14	0.07	0.13	0.02	0.13	0.02	0.13	0.09	0.13
Age	0.39*	0.11	0.43*	0.11	0.37*	0.11	0.42*	0.11	0.38*	0.11	0.42*	0.11
Intercept	–0.16*	0.06	–0.07	0.08	–0.06	0.06	0.09	0.05	–0.20*	0.08	0.35*	0.06
Linear slope	–2.80*	1.21	–2.78	1.63	–7.78	5.42	1.11*	0.49	–	–	–	–
BIC	24108.889		25357.204		23596.88		22090.428		20424.005		24317.242	
χ^2^	110.727*		183.331*		53.930*		46.065*		885.881*		38.220*	
CFI	0.82		0.56		0.96		0.97		0.50		0.97	
RMSEA	0.11		0.14		0.07		0.06		0.26		0.04	
SRMR	0.09		0.13		0.07		0.07		0.26		0.08	

*Note.* AARC = awareness of age-related change; BIC = Bayesian Information Criterion; CFI = Comparative Fit Index; Cov. = Covariance; RMSEA = Root Mean Square Error of Approximation; SRMR = Standardized Root Mean Square Residual; Var. = Variance; VLMT = Verbal Learning and Memory Task; NNB = Number N–Back. Awareness of age-related gains and losses was modeled simultaneously as correlated outcomes in six separate prediction models, one for each longitudinal construct. All growth models are parameterized in time–in–study with waves at 0, 5.406, and 9.360 years, centered at baseline. For each predictor, we estimated a linear latent growth model with random intercept and random slope; for Grip Strength and Loneliness, the linear slope variance was fixed to 0 for model parsimony. To capture practice effects, a retest factor was included for VLMT and Digit Symbol with loadings at 0, 1, and 1, and for NNB with a linear retest pattern (0, 1, 2). We consistently specified covariances between the intercept and slope and constrained residual variance to be equal across measurement occasions. All predictor and outcome variables were rescaled using *t*-scores (*M* = 50, *SD* = 10). Age was centered at 75 years. Sex and education variables were grand-mean centered. The model fit statistic is based on the full model with AARC predictions.

* *p* < .05.

**Table 3:** Baseline predictors of AARC (hearing function and visual acuity).

Variables	Hearing function		Visual acuity	
	Est.	*SE*	Est.	*SE*
**Prediction model**				
Sex	0.05	–	0.01	–
Education	–0.16	–	0.09	–
Intercept AARC-gains	47.05*	2.77	48.50*	2.23
Intercept AARC-losses	57.03*	3.17	56.95*	2.31
Residual variance, gains	99.58*	9.28	95.31*	7.23
Residual variance, losses	107.44*	7.77	97.63*	6.34
Cov. gains, losses	8.64	5.88	9.24	4.81
AARC-gains on				
Sex	1.64	1.08	1.24	0.91
Education	0.23	0.21	0.14	0.16
Age	–0.17	0.14	–0.14	0.12
Intercept	0.04	0.05	0.02	0.05
AARC-losses on				
Sex	0.56	1.13	0.44	0.89
Education	0.06	0.20	0.22	0.16
Age	0.68*	0.16	0.53*	0.12
Intercept	–0.06	0.06	–0.08	0.05
BIC	5281.193		7708.922	
χ^2^	25.493*		37.170*	
CFI	1.00		1.00	
RMSEA	0.00		0.00	
SRMR	0.00		0.00	

*Note*. AARC = awareness of age-related change; BIC = Bayesian Information Criterion; CFI = Comparative Fit Index; RMSEA = Root Mean Square Error of Approximation; SRMR = Standardized Root Mean Square Residual. Hearing function and visual acuity were measured at T1 only and included as time-invariant predictors; hence, no growth factors were modeled. All predictor and outcome variables were rescaled using *t*-scores. Age was centered at 75 years. Sex and education variables are grand mean centered.

* p < .05.

Supporting H3, loneliness showed the strongest association with AARC-losses compared to the other health and cognitive functioning indicators, with a 1-*SD* increase in loneliness corresponding to a 0.29-*SD* increase in AARC-losses, compared with 0.12-*SD* and 0.18-*SD* increases for recall memory and grip strength, respectively. Lower baseline levels of loneliness were also the only predictor for higher AARC-gains at T3.

## Discussion

The goal of the present study was to empirically test the distal antecedent component of the Awareness of Age-Related Change framework (Diehl & Wahl, 2010) by examining how baseline levels and 9-year changes in physical health (i.e., morbidity, grip strength, hearing loss, vision acuity), cognitive functioning (i.e., recall, working memory, processing speed), and psychosocial well-being (i.e., loneliness) predict gain- and loss-related awareness of aging. We found partial support for our first hypothesis. Poorer average functioning and steeper 9-year decline across the three domains were associated with more AARC-losses and fewer AARC-gains for several, but not all, indicators. Our findings corroborate, qualify, and extend the theoretical propositions of the AARC framework in three ways. First, within broader functional domains (e.g., cognition), some dimensions (e.g., recall) appear more closely associated with loss-related aging awareness than others (e.g., processing speed, working memory). Second, loss-related aging awareness is primarily associated with 9-year health and cognitive functioning trajectories, particularly higher morbidity and steeper declines in recall. It seems plausible that cumulative, long-term processes of health decline, rather than current health status alone, contribute to negative subjective aging experiences. Third, only loneliness was associated with gain-related aging awareness, suggesting that feeling less lonely (and not better physical health) is more characteristic of a gain-oriented view of aging.

### Cumulative health and cognitive decline as a pathway to loss-related aging awareness

Physical health and cognitive functioning were associated only with AARC-losses, but not with AARC-gains, which supports our second hypothesis. To begin with, a steeper 9-year increase in morbidity, but not baseline morbidity, predicted greater AARC-losses. Thus, the rate at which health is deteriorating seems to matter more than the absolute number of chronic conditions an individual has. Our findings fill an important gap in previous research. Sabatini and colleagues (2022) observed considerable variability in physical health changes across 20 years. They lacked statistical power to regress the slope parameters on AARC outcomes also measured in their study, but speculated that individuals with steeper health declines might show greater loss awareness–a proposition our results were able to support. We also build on Schönstein et al. (2021), who argued that it is not necessarily the severity of a single condition per se, but rather the presence of several conditions that triggers loss-related aging perceptions. Overall, these findings fit with the lifespan view that loss processes increasingly dominate experience in later life (Baltes, 1987; Baltes & Smith, 2003). In this vein, supporting older adults in managing health conditions early through adequate access to resources and structural support for disease management could help slow the accumulation of morbidity over time (which would be desirable in itself), thereby reducing the likelihood of developing more negative views of aging. We note that the absence of a morbidity level effect may be attributable in part to limited variability in morbidity at T1. Most participants were relatively healthy back then, with only about 27% reporting two or more chronic conditions.

In contrast to morbidity, lower baseline grip strength was associated with heightened AARC-losses, consistent with prior cross-sectional findings (Stephan et al., 2015). Grip strength is widely used as a simple and inexpensive tool for detecting vulnerabilities in older adults (Kemala Sari et al., 2025) and could likewise indicate a higher risk of experiencing loss-focused aging awareness.

We found no support for an association between sensory functioning and AARC. One possible explanation for this is the lack of longitudinal data for these measures. Without repeated measures, we were unable to model individual change trajectories, which may be more strongly related to age awareness. It seems plausible that, as for morbidity, cumulative decline in sensory functioning (rather than baseline levels) may predict loss-related age awareness. A counterargument might be that sensory functioning is more closely tied to the proximal, immediate experience of aging. For example, in an in-depth interview study with 15 older adults aged 63–75, participants described that the initial recognition of hearing difficulties triggered a heightened focus on age-related losses, though it appeared to lessen for some as they accepted and adapted to their hearing loss (Koch et al., 2025b). Studies with intensive longitudinal assessments of sensory functioning that capture transitions between onset and adjustment periods are necessary before strong conclusions can be drawn.

Among the three cognitive domains we examined, only poorer recall was linked to heightened AARC-losses, even though all three domains declined significantly over the 9-year period. Memory difficulties, including forgetting names or struggling to retrieve words, are among the most frequently reported cognitive complaints in older adults, have direct consequences for everyday functioning (e.g., making conversation), and are commonly understood as a function of growing older (Heckhausen et al., 1989; Huang et al., 2026). Memory may therefore be strongly reflected in older adults’ AARC (Diehl & Wahl, 2010; Hoffman, 2025). This interpretation is consistent with prior research showing that memory is more consistently linked to subjective aging than other cognitive domains (e.g., Fernández-Ballbé et al., 2023; Hoffman, 2025). Hoffman (2025) further speculated that this may be because the brain regions involved in subjective aging overlap more strongly with memory-related regions than with those involved in other cognitive tasks. Or, completing a recall memory test may itself be a source of heightened stress for older adults (Geraci et al., 2018; Hughes et al., 2013) and therefore a reminder of age-related declines (Hoffman, 2025).

Our null findings for processing speed and working memory may be partially explained by the fact that people are not yet consciously aware of changes in these domains. Sabatini et al. (2022) reported that 20-year declines in processing speed predicted AARC-losses, whereas our study covered a considerably shorter 9-year interval. Age-related declines in processing speed may become noticeable in everyday life only after such extended periods. Relatedly, participants in Sabatini et al. (2022) were older at the final assessment wave (mean age of 82.5), thus entering what Baltes and Smith (2003) describe as “fourth age,” a developmental period during which cognitive decline tends to become more pronounced. Limited interindividual differences in change on the Digit Symbol task in our sample may also have hampered our ability to detect meaningful associations with AARC-losses.

### Loneliness as a key distal antecedent of loss- and gain-related aging awareness

The strongest predictor of loss-focused aging awareness, above and beyond health and cognitive markers, was higher levels of loneliness, supporting our third hypothesis. Loneliness predicts poorer late-life outcomes in part through its presumed influence on functional ability, self-rated health, and depressive symptoms (Kim et al., 2020), and can reduce motivation for self-care (Hawkley & Cacioppo, 2010). Lonely older adults might thus be less likely to adapt proactively to age-related challenges and, over time, become increasingly aware of age-related losses. This interpretation is supported by the AARC premise itself, which conceptualizes age-related awareness as a specific form of self-knowledge that incorporates evaluative information about perceived age-related changes (Diehl & Wahl, 2010). Loneliness, as an emotional and subjective experience that reflects unmet social needs and is stereotypically associated with aging (Puyané et al., 2025), may therefore feed into and contribute meaningfully to the formation of loss-focused aging awareness.

Loneliness was also the only one of the eight predictors to significantly predict AARC-gains, further supporting our third hypothesis. Psychosocial resources, such as social connectedness and meaningful relationships, therefore appear more decisive for viewing aging through a gain-focused lens than maintaining good physical health alone. This aligns with Socioemotional Selectivity Theory, which holds that as people perceive their future time as more limited, they increasingly prioritize emotionally meaningful relationships (Carstensen et al., 2003). Interventions that strengthen social connectedness could potentially support more positive subjective aging experiences via multiple pathways, among others, because loneliness is linked to a range of adverse health outcomes that themselves likely contribute to more negative age views (for example, cognitive functioning: Kim et al., 2020). At the same time, while loneliness is often presumed to increase with advancing age, recent research has shown that the risk of becoming lonely is relatively stable; what is increasing with age is the risk of staying lonely (Huxhold & Henning, 2023). It may thus be intriguing to examine whether promoting positive, socially oriented views of aging could support older adults in overcoming persistent loneliness in later life.

The present findings on the distal antecedents of AARC raise broader conceptual considerations about how loss-related awareness should be approached in intervention contexts. While reducing negative views of aging has been a central goal of intervention studies in this space (Zhu et al., 2025), it remains an open question (and important philosophical issue) whether greater awareness of “loss” is always undesirable. On the one hand, excessive focus on losses, or strong attribution of everyday difficulties to aging, may contribute to distress, negative self-perceptions of aging, or maladaptive aging experiences (e.g., Wurm et al., 2017). For example, Tingvold and colleagues (2026) recently showed that, on days when older adults perceive more AARC-losses, the negative impact of health problems on subjective age and subjective accelerated aging is amplified, and suggest that “introducing strategies to reduce loss salience on days representing higher symptom loads” (Tingvold et al., 2026) could be a useful intervention target. On the other hand, acknowledging loss-related aging experiences may (at least in some cases) help older adults to recognize and manage health challenges. Insights from metacognition research highlight that accurate self-awareness and regulation of one’s own cognitive processes is critical for maintaining everyday functioning (Flavell, 1979). AARC may operate similarly in that it may trigger a host of reactions, including self-regulation and adaptational efforts (Diehl & Wahl, 2010). For example, recent work has shown that higher awareness of age-related losses may drive engagement in response-driven, adaptive health behaviors such as future care planning (Sabatini et al., 2025) and hearing aid use and formal help-seeking for hearing difficulties (Koch et al., 2026). Thus, AARC may serve two complementary purposes for interventions: First, AARC may help identify individuals at risk for maladaptive aging experiences and emerging functional decline, thereby providing opportunities for interventions targeting underlying cognitive or health-related functioning itself. Second, in cases where some degree of decline may be unavoidable, interventions may help individuals adapt more constructively to age-related changes through coping strategies, compensatory mechanisms, and more balanced interpretation of aging experiences. In this sense, the goal is not to eliminate realistic awareness of age-related losses, but rather to support adaptive adjustment to such changes (Dutt et al., 2018). Future studies should also take into account whether and how interventions targeting AARC may alter the conceptual meaning or predictive validity of AARC scores over time, and identify the conditions under which lowering or raising loss-related awareness is most likely to support positive adaptation to aging.

### Study limitations

In closing, we note limitations of our study design, sample, and measures. First, AARC was assessed only once (at T3). As a consequence, we were unable to model change trajectories over time or link changes in health and psychosocial well-being to changes in AARC. This also meant we could not assess possible reciprocal associations between health and AARC (Kornadt et al., 2020). Future longitudinal studies incorporating lagged or cross-lagged panel designs are needed to examine these reciprocal associations more directly. Second, measurement occasions were spread 4–5 years apart, which may have been too long to capture more fine-grained dynamics that evolve over weeks or months. In a similar vein, with only three measurement occasions, we were limited to modeling linear-only prediction models, though several predictors, particularly cognitive functioning, follow non-linear trajectories in later life. Future long-term longitudinal research with more frequent assessments will allow a clearer depiction of within-person (co-)developmental processes of both health and psychosocial well-being and their longitudinal associations with aging awareness.

Third, morbidity and grip strength data were available for only about half of the original sample by T3, and it is quite possible (and perhaps even likely) that relatively more of the initially less healthy participants dropped out of the study. As a result, our longitudinal estimates could underrepresent individuals with higher morbidity or declining grip strength. An additional limitation concerns our operationalization of episodic recall memory. The primary analyses used the VLMT total learning score across five trials, a composite measure that captures not only immediate recall but also learning across repeated exposures and practice-related gains. This score is widely used as an indicator of episodic memory (e.g., Düzel et al., 2018) and therefore allows comparison with prior work using the same convention. However, we acknowledge that the composite score we used may be partially affected by learning-related processes, which could overestimate recall ability. Supplementary analyses using first-trial recall only yielded less conclusive findings, partly because these data required simplified model specifications (Table S3). While speculative, one explanation could be that the learning component captured across repeated trials (rather than immediate recall alone) is more closely tied to aging awareness. At the same time, as with other cognitive abilities such as processing speed, individuals may not be fully aware of changes in their own ability to learn through repetition. Future studies specifically designed to distinguish immediate recall, learning efficiency across trials, and effects of repeated testing over time (e.g., through experimental manipulation of retest exposure) are needed to disentangle which of these cognitive processes are most strongly linked to AARC.

Participants were relatively well-educated older adults with above-average health living in the greater area of Berlin, which limits generalizability to populations with different socioeconomic or cultural backgrounds. Because contextual and cultural influences play an important role for subjective aging experiences (Wahl & Gerstorf, 2018), future research examining antecedents of views of aging in more culturally diverse samples is needed before strong conclusions can be drawn.

Finally, we relied on the two global gains and losses subscales rather than their five subdomains. We made this decision to allow for better comparability with previous research (Kaspar et al., 2022; Sabatini et al., 2022) and to avoid estimating a large number of additional models. As a natural evolution of this work, it would be intriguing to see future studies that also consider domain-specific AARC. Future research could also incorporate additional antecedents of AARC that have previously been linked to these outcomes, including stress, coping strategies, and control beliefs that likely influence ways an individual manages age-related changes (Diehl & Wahl, 2010).

## Conclusions

In the current study, we empirically tested the distal antecedent component of the Awareness of Age-Related Change framework (Diehl & Wahl, 2010). We moved beyond subjective health measures used in earlier reports and leveraged nine years of longitudinal data. Our findings provide new evidence of how developmental changes in health, cognitive functioning, and loneliness contribute to AARC-gains and AARC-losses. Health and cognitive declines (particularly increasing morbidity and declining recall) are tied to loss-related aging awareness, whereas lower levels of loneliness were uniquely linked to gain-related aging awareness. Together, these results extend the current theoretical and empirical understanding of the AARC framework and highlight that maintaining good physical and cognitive health alone is not enough to keep a gain-oriented view of aging. Combating loneliness in older adulthood seems equally critical for supporting well-being and, as our findings suggest, more positive subjective aging experiences in later life.

## Supplementary Material

gbag170_Supplementary_Data

## Data Availability

The data presented in this study are available from the BASE-II office; see https://www.base2.mpg.de/7549/data-documentation (Max Planck Society, 2025) for details. To that end, we have established procedures over the past 10+ years that we have successfully implemented. We are not in a position to make the data publicly available due to concerns regarding research participants’ privacy and consent. The study was preregistered on Open Science Framework (https://osf.io/j3sha), where the hypotheses, analysis plan, measures, sample-size rationale, and statistical analysis code are documented, and all analyses were conducted as specified in the preregistered protocol.
