# Supplementary material for: Health, cognitive, and psychosocial trajectories over nearly a decade as distal antecedents of awareness of age-related change in old age
Source: J Gerontol B Psychol Sci Soc Sci. 2026 Aug 20;81(11):gbag170. doi: 10.1093/geronb/gbag170 (PMC13654195; doi:10.1093/geronb/gbag170)
Supplement: Supplementary file 1 [file gbag170_supplementary_data.pdf]

The Journals of Gerontology: Series B, Psychological Sciences and Social Science
Supplementary Material: **Koch et al. Health, cognitive, and psychosocial trajectories over nearly a decade as distal antecedents of awareness of age-related change in old age.**

Contents

Supplementary Methods 2

Supplementary Table 1. Descriptive statistics and correlation coefficients between study variables 3

Supplementary Table 2. Univariate time-in-study latent growth curve models for health-predictor variables 4

Supplementary Figure 1. Individual trajectories for health-related predictor variables 5

Supplementary Table 3. Additional analyses: Linear latent growth curve models (time-in-study) using the first VLMT trial to predict awareness of age-related gains and losses..........................6

Supplementary Methods

Additional description of the N-Back task and Digit Symbol Substitution Test (DSST) and the instructions used.

N-Back task (NNB). In the Number-N-Back task (NNB) task, sequences of three single-digit numbers (ranging from 0 to 9) were presented sequentially across three horizontally aligned cells, followed by the next sequence of three digits. This was repeated 30 times. Participants made two-choice decisions about whether the current stimulus matched the one presented three steps earlier. After four practice trials (30 runs each), participants completed six test trials (30 runs each). Responses were made via button-box presses with the left and right index fingers. The mean accuracy across all stimuli was used as the performance score. Higher accuracy indicates better working memory.

Digit Symbol Substitution Test (DSST). The Digit Symbol Substitution Test (DSST), a subtest from the Wechsler Adult Intelligence Scale-Revised (Wechsler, 1955), required participants to match symbols to digits based on a provided key, under time pressure. Within 90 seconds, participants have to write as many corresponding symbols as possible for a sequence of digits ranging from 1-9. The test score is derived from the number of correct symbol entries, with a penalty for wrong answers (score = total - wrong). Higher scores represent better processing speed.

Supplementary Table 1. Descriptive statistics and correlation coefficients between study variables

Variables	1	2	3	4	5	6	7	8	9	10	11	12	13
1. Awareness of age-related losses T3 (5-25)	–												
2. Awareness of age-related gains T3 (5-25)	.05	–											
3. VLMT T1 (8-75)	–.07	.03	–										
4. NNB T1 (0-1)	.01	.01	.28*	–									
5. Digit Symbol T1 (20-78)	–.04	.05	.23*	.38*	–								
6. Morbidity T1 (0-8)	.09*	.04	–.02	–.03	–.08*	–							
7. Grip Strength T1 (16-65)	–.05	–.07	–.05	.07	–.09*	.07	–						
8. Loneliness T1 (1-5)	.21*	–.07	.03	.01	–.04	.02	–.03	–					
9. Hearing Function T1 (2-50)	–.02	.02	–.12*	.04	–.14*	.06	–.01	–.03	–				
10. Visual Acuity T1 (0-1.25)	–.13*	.03	.07	.11*	.09*	–.03	.16*	.04	.02	–			
11. Age T3 (69-91)	.19*	–.05	–.11*	–.08*	–.10*	.02	–.14*	.02	.16*	–.35*	–		
12. Education in years (7-18)	.02	.05	.23*	.14*	.09*	.02	.15*	–.04	.03	.09*	–.03	–	
13. Sex (0-1)	.01	.04	.13*	–.06	.16*	–.11*	–.81*	–.06	–.10	–.15*	.06	–.19*	–
M	12.8	18.2	45.2	0.76	44.3	0.98	35.1	1.5	19.1	1.0	79.2	14.5	
SD	3.5	3.1	12.7	0.18	10	1.2	9.6	0.6	8.4	0.27	3.5	2.9	

Note. VLMT = Verbal Learning and Memory Task. Apart from sex, for which Spearman correlation was used, all coefficients reflect Pearson correlation coefficients.
*p <.01.

Supplementary Table 2. Univariate time-in-study latent growth curve models for health-predictor variables

Variables	VLMT		NNB		Digit Symbol		Morbidity		Grip Strength		Loneliness	
	Est.	SE	Est.	SE	Est.	SE	Est.	SE	Est.	SE	Est.	SE
**Growth model**												
Mean intercept	49.99*	0.38	50.02*	0.38	49.83*	0.39	49.96*	0.39	49.94*	0.40	50.00*	0.38
Mean Linear slope	0.39*	0.15	–3.87*	0.16	0.38*	0.11	1.15*	0.11	–2.14*	0.08	0.13	0.13
Mean Quadric slope	–0.07*	0.02	0.44*	0.02	–0.08*	0.01	–0.03*	0.01	0.14*	0.01	0.01	0.01
Var. intercept	55.03*	5.12	52.80*	6.29	76.79*	5.62	86.13*	9.41	92.15*	4.78	62.13*	8.54
Var. linear slope	0.42*	0.10	0.39*	0.15	0.12	0.07	1.20*	0.16	0.11	0.06	0.18	0.11
Var. quadratic slope	0.00	0.00	0.00	0.00	0.00	0.00	0.00	0.00	0.00	0.00	0.00	0.00
Cov. Intercept, linear slope	–1.10*	0.56	–2.01*	0.79	–0.002	0.39	2.66*	0.65	–1.28*	0.39	0.28	0.62
Cov. Intercept, quadratic slope	0.00	0.00	0.00	0.00	0.00	0.00	0.00	0.00	0.00	0.00	0.00	0.00
Cov. linear and quadratic slope	0.00	0.00	0.00	0.00	0.00	0.00	0.00	0.00	0.00	0.00	0.00	0.00
Residual variance	39.84*	2.96	70.23*	4.50	22.84*	2.94	16.984*	1.66	11.56*	1.19	36.28*	4.21

Note. VLMT = Verbal Learning and Memory Task. NNB = Number N–Back. All models use time-in-study as the time metric, centered at baseline (0, 5.406, 9.360). For each predictor, we estimated a quadratic latent growth model with a fixed-effect intercept, linear slope, and quadratic slope, and a random intercept and random linear slope. The variance of the quadratic slope factor was fixed to zero for all predictors (see Table S1); consequently, covariances involving the quadratic slope were fixed to 0. Variables are reported as T-scores (mean=50, SD=10).
*p <.05.

Supplementary Figure 1. Individual trajectories for health-related predictor variables

VLMT=Verbal Learning and Memory Task. All variables were rescaled to T–scores (mean=50, SD=10). The x-axis uses chronological age (years).

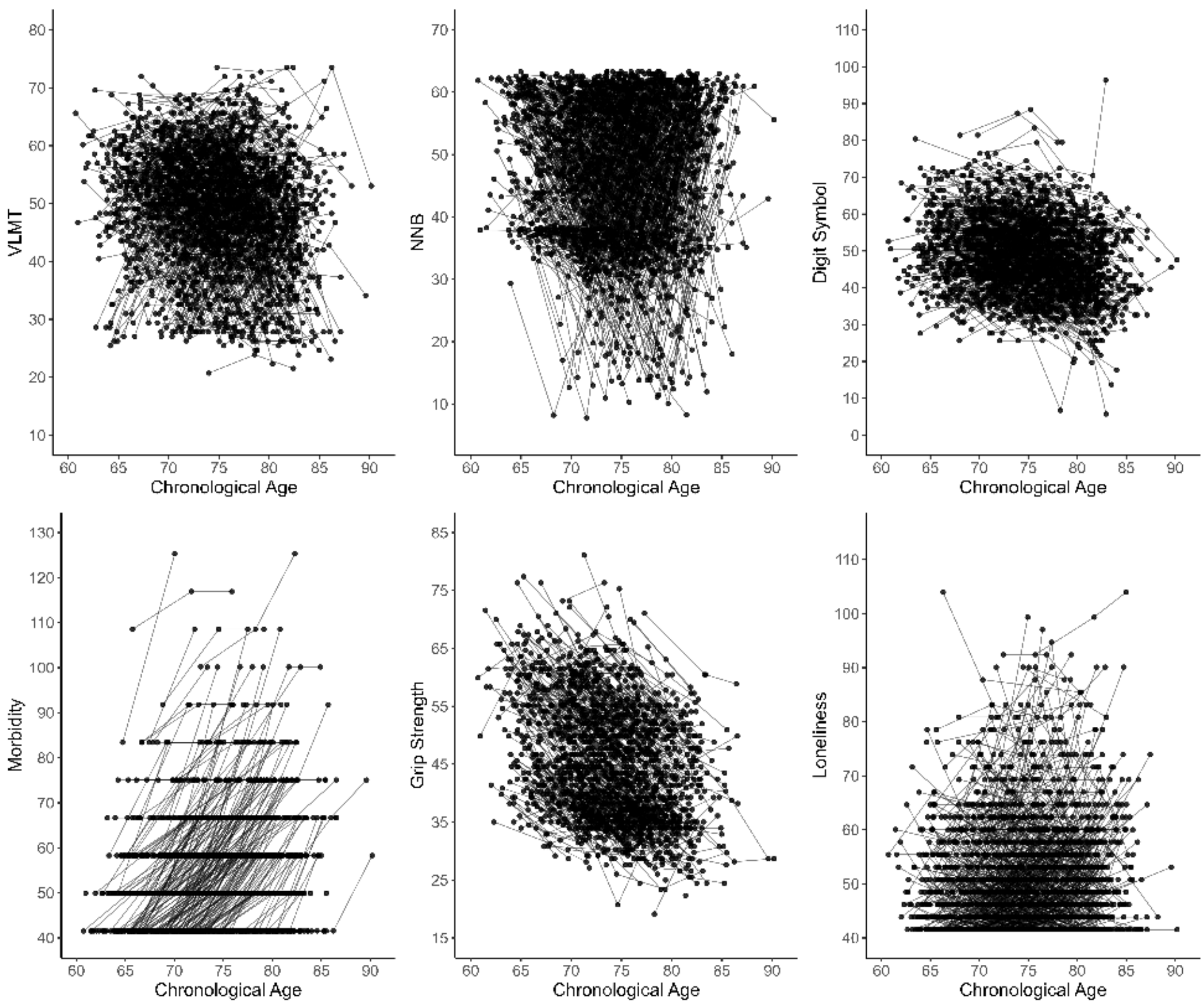

Supplementary Table 3. Additional analyses: Linear latent growth curve models (time-in-study) using the first VLMT trial to predict awareness of age-related gains and losses

Variables	VLMT Est.	VLMT SE
**Growth model**		
Mean intercept	47.93*	0.28
Mean slope	–	–
Retest	–	–
Var. intercept	32.99*	4.10
Var. slope	–	–
Cov. Intercept, slope	–	–
Residual variance	42.20*	2.92
**Prediction model**		
Sex	0.001	–
Education	–0.01	–
Age	–5.13	–
Intercept AARC-gains	47.92*	3.94
Intercept AARC-losses	52.90*	4.15
Residual variance, Gains	99.51*	6.39
Residual variance, Losses	97.81*	5.5
Cov. Gains, Losses	6.24	4.30
AARC-gains on		
Sex	1.10	0.79
Education	0.23	0.14
Age	–0.16	0.10
intercept	0.03	0.08
linear slope	–	–
AARC-losses on		
Sex	0.01	0.77
Education	0.02	0.14
Age	0.45*	0.11
intercept	–0.01	0.09
linear slope	–	–
BIC	20466.380	
χ^2	35.947*	

Note. We conducted this supplementary analysis using performance on the first VLMT trial rather than the sum score across all five trials to address concerns about learning and practice-related processes. However, the original latent growth model did not converge, necessitating estimation of a simplified intercept-only model.
